# Sensory Processing Modalities and Their Associations With Academic Achievement in Autism and Attention Deficit/Hyperactivity Disorder

**DOI:** 10.1007/s10803-025-07185-0

**Published:** 2025-12-29

**Authors:** Jennifer C. Bullen, Lindsay Swain Lerro, Tessa Hesse, Matthew C. Zajic, Nancy McIntyre, Peter Mundy

**Affiliations:** 1 Department of Experimental Psychology, University of Oxford, Anna Watts Building, Oxford OX2 6GG, UK; 2 The Swain Center for Listening, Communication and Learning, Santa Rosa, CA, USA; 3 Forward Thinking Kids, Chicago, IL, USA; 4 Intellectual Disability/Autism Program, Health and Behavior Studies Department, Teachers College, Columbia University, New York, NY, USA; 5 School of Communication Sciences and Disorders, College of Health Professions and Sciences, University of Central Florida, Orlando, FL, USA; 6 School of Education, University of California, Davis, Davis, CA, USA; 7 MIND Institute, University of California Davis, Sacramento, CA, USA

**Keywords:** Autism spectrum disorder, Academic achievement, Attention deficit/hyperactivity disorder, Sensory processing

## Abstract

**Background:**

Atypical sensory processing is common to both autistic children and children with attention deficit/hyperactivity disorder (ADHD). Some studies also suggest that atypical sensory processing may be related to academic achievement in both groups of children. However, these studies have primarily focused on overall patterns of sensory processing, as opposed to modality-specific processing (e.g., auditory), and has relied on parent report for both sensory processing and academic achievement measures. The aim of this study was to investigate whether the processing of specific sensory modalities is related to direct measures of academic achievement in autistic, ADHD, and neurotypical children.

**Method:**

74 autistic children (22 with a co-occurring diagnosis of ADHD), 34 children with ADHD, and 40 neurotypical children between the ages of 10–18 participated in this study. Parents completed reports of sensory processing, and autism and ADHD symptoms. Children completed measures of auditory processing, reading and math achievement, and IQ.

**Results:**

Autistic and ADHD children showed similar levels of atypical sensory processing, and varied patterns of associations between atypical auditory processing and academic achievement were observed in both groups.

**Conclusions:**

Sensory processing differences may impact the academic learning of both autistic and ADHD school-aged children. As neurodivergent children are integrated into general education classrooms, it is important to understand how environmental factors may impact learning opportunities for these individuals.

Difficulty with sensory processing is included as a diagnostic criterion of autism (American Psychiatric Association, 2013; Tomchek & Dunn, 2007) but it is not unique to autism. Children with attention deficit/hyperactivity disorder (ADHD) also present with difficulties in sensory processing (Dunn & Bennett, 2002; Ghanizadeh, 2011; Yochman et al., 2004). Significant difficulty with sensory processing can negatively impact the daily living skills and quality of life of children (Jasmin et al., 2009; St. John et al., 2022). One especially significant context for these negative impacts may be classroom learning.

Autistic and ADHD children are often served in general education classrooms under the Individuals with Disabilities Education Act (IDEA; Irwin et al., 2022). General education classrooms can be complex sensory environments that may be especially difficult for children who struggle with sensory processing (Hanley et al., 2017; Jones et al., 2024; Mallory & Keehn, 2021). Unpredictable loud noises like school bells, visually distracting classroom displays, and fluorescent lighting may disrupt learning by increasing anxiety or other emotional responses (Jones et al., 2020, 2024; Mallory & Keehn, 2021) or by disrupting attention, leading to distractibility and poor information processing (Dellapiazza et al., 2018; Liss et al., 2006; Moore et al., 2010). It is also the case that many autistic school-aged children without co-occurring intellectual disabilities and children with ADHD experience problems in learning and academic achievement in general education classrooms (Armstrong-Gallegos & Nicolson, 2024; Bullen et al., 2020; Butera et al., 2020; Kim et al., 2018; McIntyre et al., 2017). Parents of autistic and ADHD children report negative experiences related to academic achievement and a lack of support from schools (Classi et al., 2012; Keen et al., 2016).

Consequently, several research groups have examined the hypothesis that sensory processing difficulties negatively impact the classroom on learning of autistic or ADHD students (Armstrong-Gallegos & Nicolson, 2024; Ashburner et al., 2008; Butera et al., 2020; Davis et al., 2009; Jones et al., 2020). All of these studies provide some evidence consistent with this hypothesis, albeit there is somewhat stronger evidence for autistic students (Armstrong-Gallegis & Nicholoson, 2024; Butera et al., 2020). Nevertheless, there are several important methodological issues that warrant attention. First, three of the studies had modest samples of parent and teacher reports (10 participants or fewer with a diagnosis of autistm or ADHD; Armstrong-Gallegos & Nicolson, 2024; Ashburner et al., 2008; Butera et al., 2020) and one had a moderate sample of 59 parent or teacher reports (Jones et al., 2020). Second, none of the studies included a standardized direct measure of academic achievement or sensory processing. Instead, they relied on parent report measures of sensory processing and academic achievment. This may be problematic as sensory processing difficulties may not always be perceptible to others. Parent or teacher report of a child’s sensory processing difficulties may not be representative of their own experiences (Keith et al., 2019). Further, report measures focus on aspects of sensory processing that relate to difficulties in modulation in which there may be a mismatch between the intensity of the sensory stimuli and the response. Whereas direct measures of sensory processing may be able to identify differences in discrimination, which may relate to difficulties perceiving similarities and differences amongst sensory stimuli (Miller et al., 2007).

A third issue is that many of these studies examined measures of general styles of atypical sensory processing across sensory modalities, such as hypo- or hyper-sensitivity to stimuli, rather than atypical processing in specific modalities. These studies have been based on Dunn’s Measurement Model of Sensory Processing (Dunn, 1997, 1999, 2014) of low registration, sensory seeking, sensory sensitivity, and sensory avoiding categories of sensory processing, which collapse observations across sensory modalities. However, the full version of the Dunn’s Sensory Profile (2014) also provides measures of modality-specific sensory processing. This may be valuable because classroom learning may place more task demands on certain sensory modalities, such as auditory, visual, and tactile processing as compared to others, such as olfactory or taste processing.

Indeed, research suggests that autistic children, as well as some children with ADHD, may experience specific vulnerability to atypical processing in these modalities (Leekam et al., 2007). Auditory processing problems, especially processing filtering, audio-visual integrations and processing speech in complex sound fields can be difficult for some autistic individuals (Alcantara et al., 2004; Ashburner et al., 2008; DePape et al., 2012; Groen et al., 2009). In this regard, Jones et al. (2020) observed that parent and teacher reports of negative auditory experiences (i.e., fire alarms or other loud and unpredictable noises) had the largest negative association with their observations of learning in autistic students, followed by tactile and visual processing differences. Ashburner et al. (2008) also reported that auditory filtering and sensory under-responsiveness/seeking predicted teacher reported academic achievement among autistic students, wherein worse auditory filtering skills and more sensory under-responsiveness/seeking behaviors were associated with poorer academic performance. Autistic children may attend to or perceive tactile input longer than neurotypical children (Puts et al., 2014) and touch processing is rated as one of the most affected in the classroom by autistic teenagers (Howe & Stagg, 2016) and by observers (Fernández-Andrés et al., 2015). Other studies also indicate that autistic students display differences in social visual attention (Sasson, 2006) and non-social attention problems (McLaughlin et al., 2021) that interfer with academic task engagement (Zajic et al., 2020).

Auditory processing difficulties may also be especially common in individuals with ADHD (Bijlenga et al., 2017; Dellapiazza et al., 2020), and these difficulties may increase with age (Cheung & Siu, 2009). Indeed, it can be difficult to distinguish symptoms of ADHD from symptoms of an auditory processing disorder (APD; Cacace & McFarland, 2006; Chermak et al., 1999). There is little empirical data on the classroom impact of modality-specific sensory experience of students with ADHD. Nevertheless, children with ADHD may also experience difficulties with visual and touch processing as well (Little et al., 2018; Puts et al., 2017). Moreover, Davis et al. (2009) reported that sensory-motor functions (including auditory, visual, and tactile discrimination abilities) were estimated to account for 65% of the variance in academic achievement and 31% of cognitive processing abilities in a sample of students with ADHD. Thus, it is likely that students with ADHD may also experience disruptions to learning due to challenges with sensory processing.

## The Current Study

Based on the previous literature, the current study was designed to investigate the relationships between specific sensory modalities and individual difference in performance on standardized measures of academic achievement in children with autism, ADHD, or neurotypical (NT) development. This study examines group differences in these relationships to understand if autistic children and children with ADHD experience similar patterns of difficulties in sensory processing. This study employed a parent-report measure of sensory processing as well as a direct measure of auditory processing to compare parent and direct data on sensory processing. Thus, the study had the following three aims:

1. Test the hypothesis that autistic children and children with ADHD would differ from NT children development on parent reports of auditory, visual, and tactile sensory processing as well as the direct measure of auditory processing when controlling for possible differences in full-scale IQ (FIQ).
2. Test the hypothesis that differences in sensory processing would be related to academic achievement in the autism and ADHD groups and examine the data for possible clinical group differences in the pattern of these associations.
3. Test the hypothesis regarding the extent to which atypical sensory processing accounts for variance in FIQ in the autism and ADHD groups of children.

## Method

### Participants

The study included 74 autistic children (*M*_*AGE*_ = 12.59 years, *SD* = 2.10; *M*_*FIQ*_ = 97.78, *SD* = 14.48), 34 non-autistic children with ADHD (*M*_*AGE*_ = 13.17 years, *SD* = 2.27; *M*_*FIQ*_ = 99.65, *SD* = 15.94), and 40 NT children (*M*_*AGE*_ = 12.69 years, *SD* = 2.26; *M*_*FIQ*_ = 115.00, *SD* = 13.37). Internal Review Board’s approval was obtained prior to the implementation of this study. Research participants were recruited from local elementary and secondary schools with flyers describing the study. The flyers were sent to homes of students by school personnel. The identities of the students were unknown to the research staff until a parent voluntarily contacted the research team.

A child identified with autism was eligible for the study if they were between the ages of 8 and 16 years old, were able to answers questions in complete sentences, and did not have any major coexisting medical problems that caused children to be absent for long periods of the academic year, such as a seizure disorder or other neurological disorder, significant sensory or motor impairment, or a child psychiatric diagnosis other than autism, ADHD, or anxiety. Co-occurring diagnoses of ADHD and anxiety were permitted since these are common diagnoses in autistic children (Stevens et al., 2016). Of our autism sample, 22 children also had a diagnosis of ADHD, as reported by their parent. A child was included in the ADHD group if they had received a community diagnosis of ADHD, had current ADHD symptoms as measured by parent report on the Conners, and *did not* have a co-occurring diagnosis of autism. For the purposes of this study, the data of participants with a co-occurring intellectual and developmental disability (FIQ < 70) were not included in this study because of the likely differences in rate of academic achievement and education placement of this subgroup of children (Kim et al., 2018). Children were included in the NT group if they did not meet criteria for ADHD on the Conners, or autism on the ASSQ, SCQ, or SRS, and did not have any known or suspected diagnoses.

### Procedure

The participants in this study were part of a 30-month longitudinal study of academic, social, and cognitive development in autistic and ADHD children. Participants were assessed at three time points separated by 15 months (± 3 weeks) during which data were collected by trained research staff in two 2.5-h sessions separated by one-to-two weeks. At the first of two sessions, informed consent was obtained from all parents and informed assent was gathered from all children. Data used in this study were from time points 2 and 3, except for demographic information, IQ, and autism and ADHD symptomatology, which were collected at time point 1. Child performance on the direct measure of auditory processing were collected at time point 2. Parent report of sensory processing, math achievement, and reading achievement were collected at time point 3.

### Measures

#### Demographic Information

Parents reported on children’s demographic information including information about their child’s classroom placement, percentage of their day spent in general education, parent education level, learning disability information, and race/ethnicity. These data were collected at time point 1. See Table 1 for a summary of participant demographic information.

#### Autism Symptomatology

Autism symptom verification was collected with the Autism Diagnostic Observation Scale – Second Edition (ADOS-2; Lord et al., 2012). Participants in the autism and ADHD groups completed Modules 3 or 4 with a research reliable member of the study. The Social Affect (SA) and Restricted and Repeated Behavior (RRB) symptom subscales were assessed as well as the total score. In all groups, additional information on autism symptomatology was collected through parent report on the Autism Symptom Screening Questionnaire (ASSQ; Ehlers et al., 1999), the Social Communication Questionnaire (SCQ; Rutter et al., 2003), and the Social Responsiveness Questionnaire (SRS; Constantino et al., 2003). Information from the ASSQ, SCQ, and SRS was used to rule out symptom presentation in the neurotypical group. The ASSQ is a 27-item scale with high test–retest (*r* = .90) and inter-rater reliability (*r* = .79; Ehlers et al., 1999). The SCQ is a 40-item parent questionnaire with high sensitivity (0.96) and specificity (0.80) estimates. Scores of 15 and above are consistent with autism (Berument et al., 1999). The SRS is a 65-item scale with high internal consistency .97 (Constantino et al., 2000) and scores above 60 are consistent with autism diagnoses.

#### ADHD Symptomatology

Parent-report on the Conners 3 (Conners, 2008) was used to assess symptoms of ADHD in all participants. The Conners 3 yields standardized *T*-scores for measures of Inattention, Hyperactivity-Impulsivity, Executive Functions, and Learning Problems based on a normative sample of 1,200 parents of 8- to 18-year-olds. Internal consistency ranges from 0.77 to 0.97 (Conners, 2008).

#### IQ

The Wechsler Abbreviated Scales of Intelligence – Second Edition (WASI-2, Wechsler, 2011) was used to provide a measure of IQ for each participant. The WASI-2 contains four subtests—Vocabulary, Similarities, Block Design, and Matrices – that provide estimates of verbal, non-verbal, and full-scale IQ (FIQ). For the purposes of this study, FIQ was used to determine if participants met criteria for this study (FIQ > 70) and as a covariate for planned analyses. The FIQ index has established internal consistency of 0.96 (Weschler, 2011). Cronbach’s alpha coefficients were 0.89 for Vocabulary, 0.88 for Similarities, 0.87 for Block Design, and 0.92 for Matrix Reasoning in this sample (McIntyre et al., 2017)

#### Mathematics Achievement

Two subtests of the Wechsler Individual Assessment Test – Third Edition (WIAT-III; Wechsler, 2009) were used as estimates of children’s mathematical achievement. The Numerical Operations subtest provides an estimate of calculation abilities. Participants completed basic math computations independently of the test administrator. The Problem Solving subtest provides a measure of verbal problem solving ability. Participants complete untimed math problems to gauge abilities in single- and multi-step word problems, fractions, decimals, mathematical patterns, interpreting graphs, and solving problems using probability and reasoning. Due to time constraints, participants completed only the odd-numbered problems at time points 1 and 3, and even numbered problems at time point 2. Reliability estimates for WIAT-III subtests range from 0.83 to 0.97 (Wechsler, 2009).

#### Reading Achievement

Two measures from the Gray Oral Reading Tests – Fifth Edition (GORT-5; Wiederholt & Bryant, 2012) were used as estimates of children’s reading achievement. The Reading Comprehension subtest is comprised of 16 progressively more difficult passages that are read aloud by the child, followed by 5 open-ended comprehension questions. Comprehension questions are presented orally by the test administrator with the reading passages removed from view. The Reading Fluency score provides a measure of one’s speed and accuracy in reading. Fluency data is collected during the same 16 passages read for the reading comprehension subtest. Participants were scored for rate (in seconds) and accuracy (number of words read correctly) to determine a fluency score for each passage. All internal consistency for GORT-5 scores exceeds 0.90 (Wiederholt & Bryant, 2012).

#### Sensory Processing

The Sensory Profile – Second Edition (SP2; Dunn, 2014) is an 86-item parent questionnaire designed to measure the sensory behaviors and patterns across 6 modalities: auditory processing, visual processing, touch processing, movement processing, body position processing, and oral sensory processing. Data from the auditory processing, visual processing, and touch processing subscales were examined in this study. Parents rate each item on a scale of 0 (does not apply) to 5 (almost always). Higher scores reflect higher frequencies of sensory difficulties. The SP-2 was normed on a sample of 697 children aged 3 to 14 years and demonstrates strong psychometric properties with internal consistency alpha values ranging from 0.60–0.90 (Dunn, 2014).

A direct measure of auditory processing was provided by the SCAN-3. The SCAN-3 is a battery of tests used to screen and diagnose auditory processing disorder in children (SCAN-3:C; Keith, 2000) and adolescents (SCAN-3:A; Keith, 2009). For the purposes of this study, data from the three diagnostic subtest and one screening test of the SCAN-3 were examined. The screening test was the Auditory Figure-Ground: *0 dB* subtest, in which forty words are presented to either the left or right ear against a background of noise recorded at the same volume as the word. The participant is asked to repeat the words.

Three additional diagnostic tests were administered. First, Filtered Words includes forty words that are presented in either the left or right ear, with higher frequencies of sounds removed, which give the words a muffled quality. Participants are asked to repeat the word or provide a guess if they are unsure. Second, Competing Words: Directed Ear includes thirty pairs of words are presented dichotically, but in this test, participants are asked to repeat the word presented in either the left or right ear first. No credit is given for words repeated in the incorrect order. Finally, Competing Sentences includes twenty pairs of short sentences are presented dichotically. Participants are instructed to either repeat the sentence presented to either the left or right ear first.

Each child received a score for each subtest as well as the SCAN-3 Auditory Processing Composite Score, which is derived from the diagnostic subtests and the screening subtest used in this study. The SCAN:C was normed on 650 children between the ages of 5 to 11 years old. SCAN:C scores were comparable to SCAN scores with a correlation of 0.79 between the two tests (Keith, 2000).

### Data Analysis Plan

To understand group differences in parent report on the SP2 auditory, visual, and touch processing scales, a multivariate analysis of covariance (MANCOVA) was conducted with group (autism, ADHD, and NT) and included FIQ as a covariate. To understand group differences in the direct measure of auditory processing scores, we conducted a univariate analysis of covariance (ANCOVA) with the SCAN-3 Auditory Composite Score and included FIQ as a covariate. We also examined if parent-report on the SP2 Auditory Processing and the SCAN-3 Auditory Processing measures were associated by examining Pearson correlation coefficients in each of the three groups. Finally, to understand the impact of sensory processing on academic achievement, we used linear regression models with FIQ as a covariate. FIQ was entered in Model 1 and variables were added individually in subsequent models by order of their magnitude of correlation with academic achievement variables. Correlations and regressions were conducted separately for the autism, ADHD, and NT groups.

Post hoc considerations were raised regarding the subsample of participants in the Autism Group with co-occurring diagnoses of autism and ADHD. To understand if the results were influenced by the inclusion of these participants, we ran the specified regressions from above a second time, excluding individuals who had a co-occurring diagnosis of autism and ADHD.

## Results

### Group Differences in Sensory Processing

**Fig. 1:**
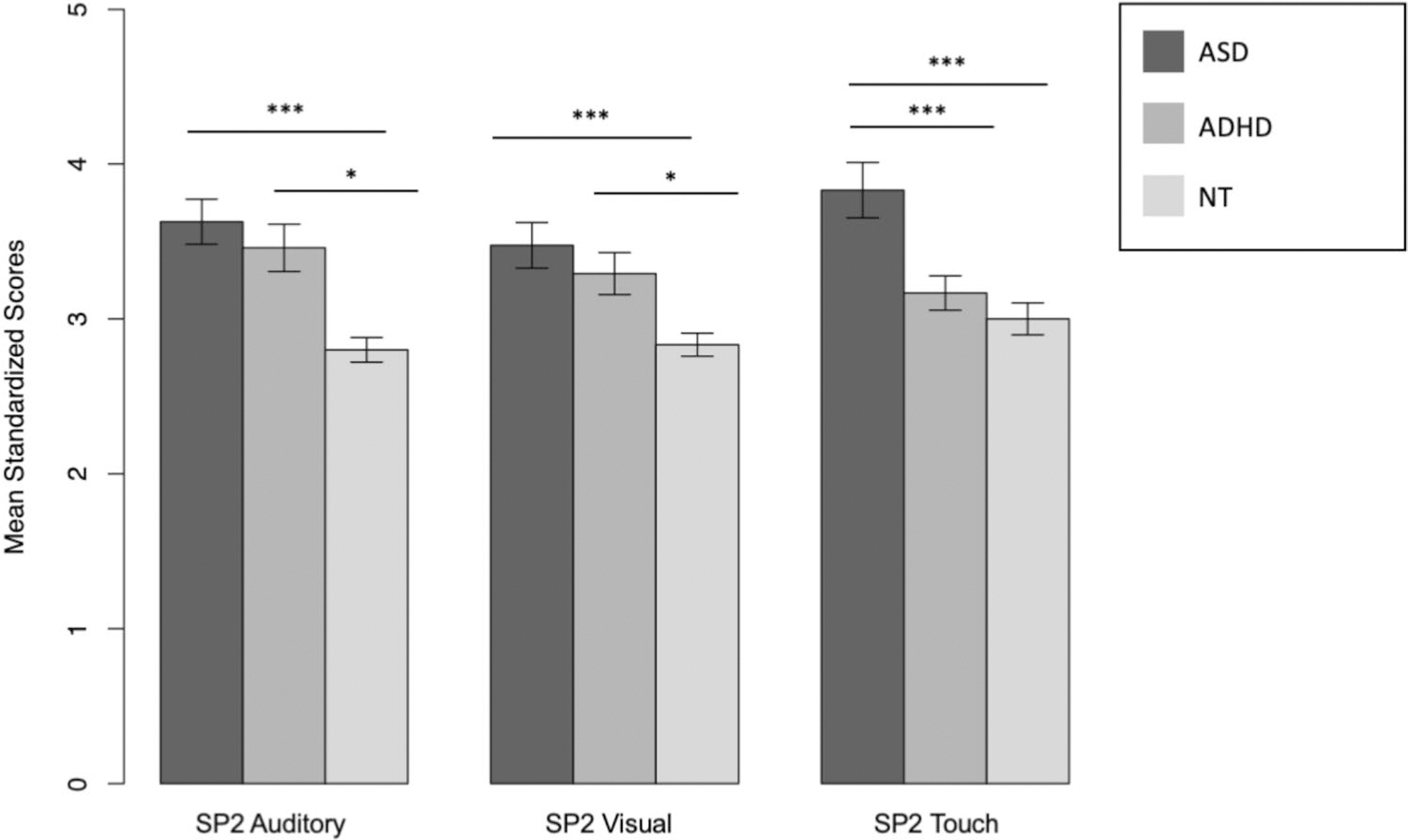
Comparisons of average Sensory Profile 2 auditory, visual, and touch measures between groups. *p < .05, **p < .01, ***p < .001. Error bars represent standard deviations. ASD = autism spectrum disorder, ADHD = attention deficit/hyperactivity disorder, NT = neurotypical, SP2 = Sensory Profile, Second Edition

First, the MANCOVA to assess group differences in the SP2 subtests (SP2 auditory, visual, and touch processing) and controlling for FIQ, indicated significant group differences, *F*(6, 214) = 7.80, Wilk’s Λ = 0.67, *p* < .001, η_p_^2^ = .14. Follow-up univariate ANCOVA for each sensory variable showed both consistent and inconsistent between-group differences (see Fig. 1).

Groups differed on SP2 auditory processing, *F*(2, 108) = 7.68, *p* = .001, η_p_^2^ = .12. Post hoc analyses with Tukey’s Multiple Comparison Test indicated that the NT group had significantly lower auditory scores (*M* = 2.80, *SD* = 0.41) than the ADHD (*M* = 3.46, *SD* = 0.78), *t*(53) = −2.54, *p* = .03, and autism (*M* = 3.63, *SD* = 0.74) groups, *t*(88) = −3.92, *p* < .001. We did not observe a statistically significant difference between the ADHD and autism groups on auditory processing, *t*(82) = 0.97, *p* = .60.

Groups also differed on SP2 visual processing, *F*(2, 109) = 9.08, *p* < .001, η_p_^2^ = .14. Post hoc analyses with Tukey’s Multiple Comparison Test indicated that the NT group had significantly lower scores (*M* = 2.83, *SD* = .38) than the ADHD (*M* = 3.29, *SD* = .69), *t*(53) = −2.68, *p* = .02, and autism (*M* = 3.47, *SD* = .75) groups, *t*(88) = −4.26, *p* < .001. Again, we did not observe a statistically significant difference between the ADHD and autism groups, *t*(82) = 1.17, *p* = .47.

Finally, groups differed on SP2 touch processing, *F*(2, 109) = 14.27, *p* < .001, η_p_^2^ = .21. Post hoc analyses with Tukey’s Multiple Comparison Test indicated that the NT (*M* = 3.00, *SD* = .52; *t*(88) = −4.80, *p* < .001) and ADHD (*M* = 3.17, *SD* = .56; *t*(82) = 3.63, *p* = .001) groups both had significantly lower touch processing sores than the autism group (*M* = 3.83, *SD* = .91). We did not observe a statistically significant group difference between the NT and ADHD groups, *t*(53) = −1.21, *p* = .50.

**Fig. 2:**
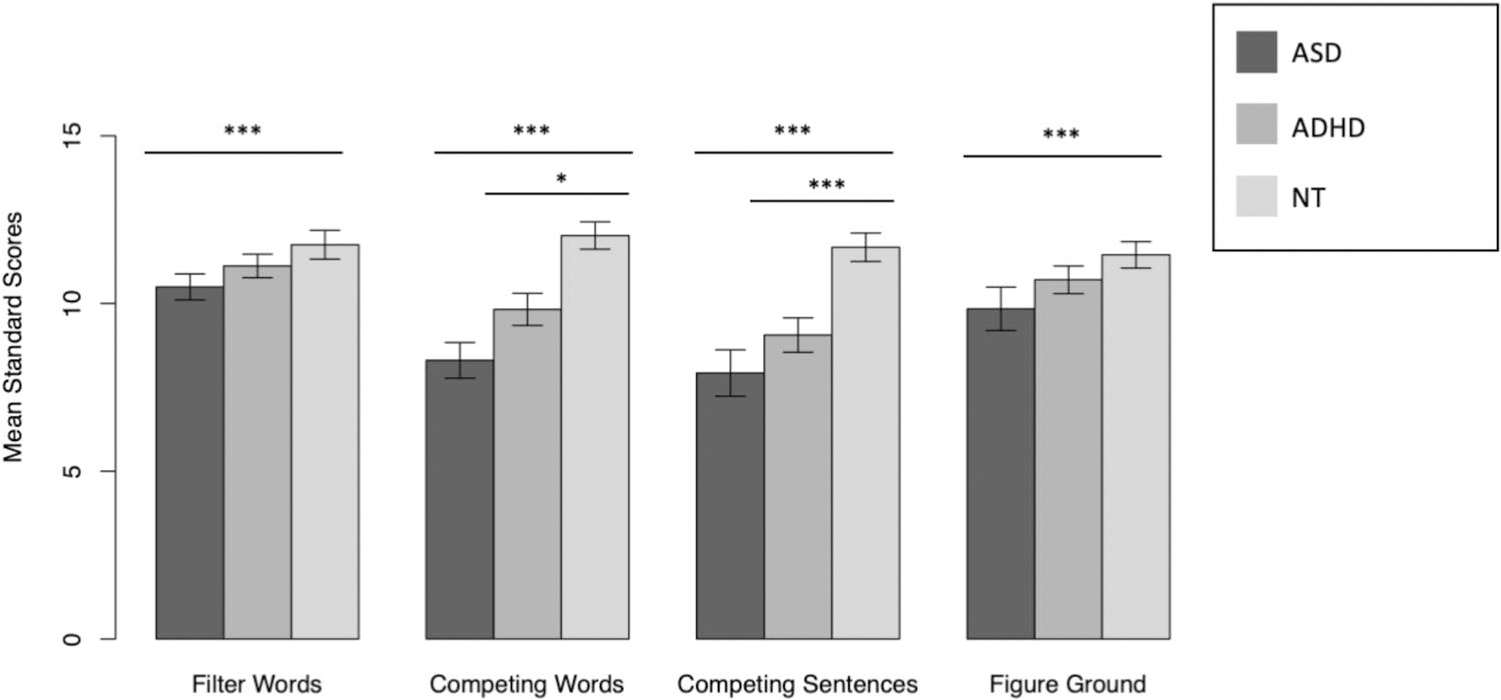
Comparisons of average SCAN-3 filter words, competing words, competing sentences, and figure ground between groups. *p < .05, **p < .01, ***p < .001. Error bars represent standard deviations. ASD = autism spectrum disorder, ADHD = attention deficit/hyperactivity disorder, NT = neurotypical

Next, a univariate ANCOVA was conducted to determine diagnostic group differences in SCAN-3 Auditory Composite Scores. Groups differed on their SCAN-3 Auditory Composite Scores, *F*(2, 138) = 11.02, *p* < .001, η_p_^2^ = .14. Post hoc analyses with Tukey’s Multiple Comparison Test indicated that the NT group had higher SCAN Auditory Composite Scores (*M* = 111.26, *SD* = 10.59) indicating better auditory processing ability than the ADHD (*M* = 97.76 *SD* = 12.02), *t*(72) = 2.39, *p* = .04, and autism (*M* = 91.46, *SD* = 16.94) groups, *t*(107) = 4.66, *p* < .001, which did not differ statistically, *t*(102) = −2.08*, p* = .10 (Fig. 2). The group averages fell in the average range for the autism and ADHD groups while the average was slightly elevated in the NT group.

### Correlation of Parent Reported Auditory Processing and SCAN-3 Auditory Processing

The parent report of auditory processing on the SP2 was significantly correlated with the SCAN-3 Auditory Processing Composite Scores in the autism group only (*r* = −.39, *p* = .004; see Table 2 for all groups). Parent report was additionally correlated with the SCAN-3 Competing Words (*r* = −.39, *p* = .003) and Competing Sentences (*r* = −.42, *p* = .002) subtests of the SCAN.

### Correlations Between Sensory Processing, Academic Achievement, and IQ

Pearson correlations were conducted in each group to investigate the associations of sensory processing with academic achievement and IQ. In the autism group, SP2 Auditory, but not Touch or Visual, was associated with FIQ (*r* = −.30, *p* = .02). Each of the SCAN-3 subtests were additionally associated with FIQ (*r*s .25–.47, *ps* < .05; see Supplementary Tables). Both measures from the SP2 and SCAN-3 demonstrated weak to moderate correlations with of academic achievement. SP2 Auditory processing was negatively correlated with GORT-5 Fluency (*r* = −.39, *p* = .002) and GORT-5 Comprehension (*r* = −.32, *p* = .01). SCAN-3 Competing Words was positively associated with GORT-5 Fluency (*r* = .36, *p* = .003). SCAN-3 Filtering Words was associated with WIAT-III Problem Solving (*r* = .42, *p* < .001). SCAN-3 Competing Sentences was positively correlated with GORT-5 Fluency (*r* = .48, *p* < .001), GORT-5 Comprehension (*r* = .43, *p* < .001), WIAT-III Numerical Operations (*r* = .32, *p* < .001), and WIAT-III Problem Solving (*r* = .40, *p* = .001).

In the ADHD group, SP2 Touch (*r* = .48, *p* = .02) and SCAN-3 Competing Words (*r* = .42, *p* = .01) were the only subtests associated with FIQ. Further, only the SCAN-3 had significant moderate correlations with academic achievement in the ADHD group, parent report on the SP2 was not associated with academic achievement. Specifically, SCAN-3 Filtering Words (*r* = .37, *p* = .04) and SCAN-3 Competing Words (*r* = .48, *p* = .01) were associated with GORT-5 Comprehension. SCAN-3 Competing Sentences was associated with GORT-5 Fluency (*r* = .58, *p* < .001), GORT-5 Reading Comprehension (*r* = .71, *p* < .001), WIATIII Numerical Operations (*r* = .64, *p* < .001), and WIAT-III Problem Solving (*r* = .44, *p* = .01). For the NT group, no scores from the SP2 or the SCAN-3 were significantly correlated with academic achievement or FIQ. See the supplementary material for correlation tables for the three groups.

### Multiple Regressions of Sensory Processing, IQ, and Academic Achievement

Linear regression analyses were conducted independently for the autism and ADHD groups to assess the impact of sensory processing variables on academic achievement after controlling for FIQ. Analyses were not conducted for NT group, as significant correlation between sensory, FIQ, and academic measures were not observed.

#### Autism Group

For GORT-5 Reading Fluency, Model 1 with FIQ (β = 0.52, *p* < .001) contributed to explaining 30% of the variance in GORT-5 Reading Fluency scores, *F*(1, 59) = 27.09, *p* < .001. The inclusion of SCAN Competing Sentences (β = .25, *p* = .02) in Model 2 explained variance in addition to FIQ (β = .40, *p* = .001), Δ*R*^*2*^ = .05, *F*(1, 58) = 5.20, *p* = .02. SP2 Auditory Processing (β = −.17, *p* = .15) in Model 3 and SCAN Competing Words (β = .06, *p* = .71) in Model 4 were not significant predictors of GORT-5 Reading Fluency and did not contribute to a significant change in explained variance.

For GORT-5 Reading Comprehension, Model 1 with FIQ (β = .63, *p* < .001) contributed to explaining 41% of the variance in GORT-5 Reading Comprehension scores, *F*(1, 66) = 45.33, *p* < .001. SCAN Competing Sentences (β = .15, *p* = .17) in Model 2 and SP2 Auditory Processing (β = −.17, *p* = .17) in Model 4 were not significant predictors of GORT-5 Reading Comprehension scores and did not contribute to a significant change in explained variance. See Table 3 for a summary of regression findings for the reading variables.

For WIAT-III Numerical Operations, Model 1 with FIQ (β = .80, *p* < .001) contributed to explaining 42% of the variance in WIAT-III Numerical Operations scores, *F*(1, 68) = 49.28, *p* < .001. In Model 2, SCAN Competing Sentences (β = .04, *p* = .74) was not a predictor of WIAT-III Numerical Operations scores and did not contribute to a significant change in explained variance.

For WIAT-III Problem Solving, Model 1 with FIQ (β = .75, *p* < .001) contributed to explaining 55% of the variance, *F*(1, 61) = 75.12, *p* < .001. In Model 2, SCAN Filter Words (β = .25, *p* = .006) contributed to explaining variance in addition to FIQ (β = .69, *p* < .001), Δ*R*^*2*^ = .06, *F*(1, 60) = 8.15, *p* = .006. SCAN Competing Sentences in Model 3 (β = −.07, *p* = .49) and SCAN Auditory Figure Ground (β = .001, *p* = .99) in Model 4 were not significant predictors of WIAT-III Problem Solving and did not contribute to a significant change in explained variance. See Table 4 for a summary of regression findings for the mathematics variables.

The above regressions were run again on a subset of the Autism Group, which excluded those who had a co-occurring diagnosis of autism and ADHD (N = 22). The main effects reported above were significant in these additional analyses, suggesting that this subgroup is not driving the results. Tables reporting regression findings are included in the supplementary materials.

#### ADHD Group

For GORT-5 Reading Fluency, Model 1 with FIQ explained (β = .39, *p* = .03) 16% of the variance in scores, *F*(1, 28) = 5.28, *p* = .03. In Model 2, SCAN Competing Sentences (β = .49, *p* = .01) but not FIQ (β = .18, *p* = .27) explained variance in GORT-5 Reading Fluency Scores, Δ*R*^*2*^ = .22, *F*(1, 27) = 8.85, *p* = .01.

For GORT-5 Reading Comprehension, Model 1 with FIQ (β = .67, *p* < .001) explained 47% of the variance in scores, *F*(1, 28) = 25.00, *p* < .001. In Model 2, SCAN Competing Sentences (β = .49, *p* < .001) explained additional variance to FIQ (β = .46, *p* < .001), Δ*R*^*2*^ = .21, *F*(1, 27) = 17.98, *p* < .001. SCAN Competing Words (β = .04, *p* = .77) in Model 3 was not a significant predictor and did not contribute to a significant change in explained variance. See Table 5 for a summary of regression findings for the reading variables.

For WIAT-III Numerical Operations, Model 1 with FIQ (β = .69, *p* < .001) explained 50% of the variance in scores, *F*(1, 28) = 27.66, *p* < .001. The addition of SCAN Competing Sentences (β = .41, *p* = .003) in Model 2 explained variance in addition to FIQ (β = .52, *p* < .001), Δ*R*^*2*^ = .14, *F*(1, 27) = 10.75, *p* < .001.

For WIAT-III Problem Solving, Model 1 with FIQ (β = .77, *p* < .001) explained 63% of the variance in scores, *F*(1, 28) = 47.97, *p* < .001. SCAN Competing Words (β = .13, *p* = .32) in Model 2 and SCAN Competing Sentences (β = .10, *p* = .45) in Model 3 were not a significant predictor and did not contribute to explaining additional variance. SCAN Auditory Figure Ground (β = .25, *p* = .04) in Model 4 was a significant predictor but did not lead to statistically significant changes in explained variance, Δ*R*^*2*^ = .07, *F*(3, 25) = 2.12, *p* = .12. See Table 6 for a summary of regression findings for the mathematics variables.

## Discussion

This study sought to understand how sensory processing difficulties relate to academic outcomes in autistic children and children with ADHD. First, we investigated the sensory processing profiles of autistic and ADHD children compared to their NT peers. As expected, the autism and ADHD groups had higher scores on the auditory and visual processing measures of the SP2 than the NT group. This indicates that parents of children in the autism and ADHD groups reported more difficulties with auditory and visual processing than parents of children in the NT group. Only touch processing on the SP2 differed between children in the autism and ADHD groups, wherein parents of autistic children reported more difficulty with touch processing than ADHD or NT parents. Similarly, the autism and ADHD groups had worse scores on the SCAN-3 Auditory Composite Score than their NT peers. These findings are consistent with previous research indicating that both autistic children and children with ADHD have difficulty with sensory processing compared to their NT peers (Dunn & Bennet, 2002; Ghanizadeh, 2011; Tomcheck & Dunn, 2007).

A primary aim of the current study was to test the hypothesis that difficulty with sensory processing is related to academic outcomes in school-aged autistic children (Ashburner et al., 2008; Butera et al., 2020; Jones et al., 2020) and children with ADHD (Davis et al., 2009). As part of this goal, we wished to examine the hypothesis that autistic children and children with ADHD may show different corollaries of sensory processing difficulties (Little et al., 2018), despite displaying similar levels of sensory difficulties (Dellapiazza et al., 2018). In the autism group, both the SCAN-3 and SP2 measures of auditory processing demonstrated significant correlations with reading (GORT-5) and mathematics (WIAT-III). Interestingly, neither SP2 parent report of visual nor touch processing were associated with academic outcomes in the autism group. In the ADHD group, only the SCAN-3 demonstrated significant correlations with reading and mathematics. However, the findings from the regression analyses indicated that for both the autism and ADHD groups, only the SCAN-3 measures contributed unique variance. Thus, both groups demonstrated a relationship between auditory processing difficulties and academic achievement outcomes. This raises the possibility that hyper- or hypo-sensitivity to sounds and the filtering of background noise may impact academic achievement in the autism and ADHD groups. This was contrary to our hypothesis, as there were not differential contributors to academic achievement between our two groups.

Nevertheless, these findings are consistent with previous reports that have indicated that parents and teachers report auditory processing differences as having the largest negative impact on learning in autistic students compared to touch or visual processing (Jones et al., 2020). An additional study reported that auditory processing predicted teacher-reported academic achievement among autistic students (Ashburner et al., 2008). The findings from the SP2 and SCAN-3 data in this study were also similar to previous observations that autistic children display sensitivity to sounds (Jones et al., 2003; Khalfa et al., 2004) and that difficulty with the processing of speech in the presence of complex background noise may be particularly difficult (Alcantra et al., 2004; Groen et al., 2009; O’Connor, 2012). These findings are additionally consistent with previous research indicating that auditory processing difficulties are common in individuals with ADHD (Bijlenga et al., 2017; Dellapiazza et al., 2020) and auditory discrimination may contribute to variance in academic achievement (Davis et al., 2009). However, unlike previous research, we did not find evidence for the contribution of visual and tactile discrimination to differences in academic achievement (Davis et al., 2009).

Differences in the corollaries between the sensory variables (SP2 and SCAN-3) and academic achievement in our autism and ADHD groups may reflect that hyper- or hyposensitivity to sounds and filtering background noise while processing spoken language may reflect different auditory processes (Dawes & Bishop, 2009). In this study, the SP2 measured the former and SCAN-3 measured the latter. It is interesting to note that the correlation of these two measures observed in this study was moderate in the autism group, but these measures were not related in the ADHD or NT groups. This may indicate that the measures tapped into unique and common variance in auditory processes in autistic children. The unique processes may be related to the degree they reflect cortical (Chang et al., 2014) and subcortical processes (Font-Alaminos et al., 2020) associated with auditory processing in autism. Thus, it may be useful to examine both types of processes comparatively and in greater detail in future research to understand their independent or interactive roles in academic achievement. Regardless, evidence from brain and behavioral research indicate that difficulty with auditory processing is common in autism (Demopoulos & Lewine, 2016; O’Connor, 2012; Vlaskamp et al., 2017). Findings from this study suggest that this may be important to recognize in order to provide optimal assessments and educational supports for autistic students in general education classrooms. This will require a more precise understanding of how auditory processing relates to the learning and academic difficulties faced by many autistic students without intellectual abilities (Bullen et al., 2020; Kim et al., 2018; McIntyre et al., 2017). As the sample of children with ADHD is small in this study, additional research is needed to understand if these differences between parent report and direct measure are replicable. However, if this pattern of results is upheld, one possibility may be that sensory discrimination and sensory modulation are separable aspects of sensory processing in children with ADHD. Sensory processing is understudied in ADHD as compared to autism, but the findings here suggest that there is a need to understand sensory processing in these children further as it may negatively impact academic achievement. Sensory processing may be an important consideration for understanding the experiences of children with ADHD in education and being able to provide adequate classroom support.

Finally, IQ was used as a covariate in this study as we sought to investigate the role of sensory processing in academic achievement above the influence of IQ. However, there are important considerations to make regarding the relationship of sensory processing and IQ that warrant consideration. Previous research regarding the influence of IQ on sensory processing has been mixed. A few studies have suggested that IQ may explain differences in sensory processing (Baranek et al., 2006, 2013; Hulslander et al., 2004; Jasmin et al., 2009; Narzisi et al., 2022), while others have found that IQ was not related to sensory processing difficulties (Ashburner et al., 2008; Sanz-Cervera et al., 2015). Additionally, previous research has indicated that the link between sensory processing and IQ may be most apparent in children with co-occurring intellectual disabilities (Leekam et al., 2007; Werkman et al., 2022). However, our findings show that parent-reported auditory processing and the directly assessed measure of auditory processing were significantly correlated with FIQ in the autism group. The data here and elsewhere (Butera et al., 2020; Hochauser & Engle-Yeager, 2010) suggest IQ may be associated with sensory processing among autistic children without co-occurring intellectual disabilities. Nevertheless, IQ measures assess cognitive factors that can predict academic achievement (Konold & Canivez, 2010), which may provide additional evidence that sensory processing may be related to factors associated with academic achievement. Further research is needed to disentangle the impacts of sensory processing on cognitive factors and academic achievement.

### Implications

The findings here suggest that both children with ADHD and autistic children experienced difficulties with sensory processing. In both groups, aspects of auditory processing, captured by the direct measure, explained variance in academic achievement. These add to the small body of literature which suggest an overreliance on verbal instructions may not be appropriate for autistic children or those with ADHD (Ashburner et al., 2008). These children may require additional supports, such as written instructions or visual schedules, in order to engage in classroom activities alongside their neurotypical peers. It may also be the case that loud classrooms, unexpected noises, etc. may be especially distressing to some students. Difficulty with regulating this kind of sensory input could make it more difficult to engage in classroom activities. Further, it also suggests that assessing auditory processing skills may be an important part of assessing learning difficulties in neurodivergent children. The necessary supports for a child experiencing academic difficulty due to auditory processing differences may be very different from academic difficulty caused by other factors. Considering sensory processing needs in education is an important next step in providing appropriate educational supports for neurodivergent children.

### Limitations

The data in this study indicate that individual differences in auditory sensory processing may be significantly related to the academic learning problems that have been observed for many autistic school-aged children without intellectual disabilities and children with ADHD. The data indicated that individual differences in auditory sensitivity, especially with regard to the direct auditory measure, may be related to differences in academic outcomes for autistic and ADHD children. However, the unequal sample sizes of the autism and ADHD group was a limitation in this study, such that the relative power to observe significant effects was different across the group.

Further, this study includes a portion of individuals who have co-occurring diagnoses of autism and ADHD (AuDHD). At the time that this data was collected, DSM-V criteria that allowed for a dual diagnosis was relatively recent. However, there is increasing recognition of the co-occurrence of these two conditions (Leitner, 2014). The experiences of those with co-occurring diagnoses of autism and ADHD are likely different than those with a diagnosis of one or the other. Indeed, AuDHD advocates note that their experiences often differ than those of individuals with either of these diagnoses in isolation (Craddock, 2024). Research on the experience of AuDHD individuals is incredibly limited and it was not within the scope of this research, nor was the sample size sufficient, to explore the unique sensory experiences of these individuals. This continues to be a limitation of this study and autism and ADHD research more broadly. It should be noted that this study found that although autism and ADHD had different corollaries with academic achievement, there were note differences between the groups in terms of sensory processing contributors to academic achievement. However, it is possible that the increased difficulty with attention combined with characteristics of autism may interact with sensory processing difficulties differently than these would in children with only one of these diagnoses. Thus, we cannot generalize these findings to the experiences of AuDHD individuals, as their experiences are likely different. Further research is needed to understand the sensory and academic experiences of AuDHD individuals in order to understand how to best support these learners in their education.

Several limitations in the study and the interpretation of its data should also be recognized. While this study benefitted from a direct measure of auditory processing, it lacked self-reported sensory processing data, which may be key in understanding autistic individuals’ own perceptions of their sensory processing difficulties. Previous research indicates that adolescents may report greater sensory symptoms on self-report measures than their parents do about them on parent-reported measures (Keith et al., 2019). Few studies have used self-report measures of sensory processing (De la Marche, 2012), yet it will be important for future research to incorporate these measures to gauge the experiences of students more accurately in classrooms. Additional direct measures for visual and touch processing may also provide more accurate information on sensory processing and its effects in future studies of autism and ADHD.

Finally, the sample demographics are a limitation in terms of understanding the needs of diverse samples of autistic individuals. Our sample is primarily White and male without co-occurring intellectual disability (FIQ > 70). Thus, the findings may not be indicative of the experiences of a more diverse sample with regard to racial or gender identities, or the experiences of individuals with co-occurring intellectual disabilities.

## Supplementary Material

Supplementary Material

**Supplementary Information** The online version contains supplementary material available at https://doi.org/10.1007/s10803-025-07185-0.

## Figures and Tables

**Table 1:** Summary of demographic information for participants in this study

	Autism (N = 74)	ADHD (N = 34)	NT (N=40)

Age (in years)	12.59(2.10)	13.17(2.27)	12.69(2.26)
Grade	5.56(2.14)	6.25(2.18)	5.95(2.21)
FIQ	97.78(14.48)	99.65(15.94)	115.00(13.37)
SRS Total	81.92(10.59)	61.52(15.54)	45.05(8.78)
SCQ Total	21.14(7.07)	6.85(6.33)	2.38(2.18)
ASSQ Total	18.54(5.40)	8.82(7.09)	1.94(2.82)
Conners Inattention/Hyperactivity Average	73.71(12.06)	75.41(11.89)	48.41(8.73)
Sex			
Male	62 (84%)	27 (79%)	26 (65%)
Female	12 (16%)	7 (21%)	14 (35%)
Mother Education			
Some or Completed High School	3 (4%)	3 (9%)	0 (0%)
Some or Completed College	43 (58%)	22 (65%)	25 (63%)
Some or Completed Graduate School	27 (36%)	8 (23%)	13 (33%)
Missing	1 (1%)	1 (3%)	2 (5%)
Father Education			
Some or Completed High School	6 (8%)	5 (15%)	1(2%)
Some or Completed College	46 (62%)	22 (65%)	21(53%)
Some or Completed Graduate School	21 (28%)	6 (18%)	16(40%)
Missing	1 (1%)	1 (3%)	2 (5%)
Race			
Asian	3 (4%)	0 (0%)	3 (8%)
Black/African American	0 (0%)	1 (3%)	0 (0%)
Native Hawaiian/P.I	0 (0%)	1 (3%)	1 (3%)
White/Caucasian	52 (70%)	27 (79%)	32 (80%)
Decline to State	2 (2%)	0 (0%)	0 (0%)
Other	3 (4%)	2 (6%)	0 (0%)
Multiracial (inc. White)	6 (8%)	2 (6%)	2 (5%)
Multiracial (not inc. White)	1 (1%)	0 (0%)	0 (0%)
Missing	0 (0%)	0 (0%)	2 (5%)
Ethnicity			
Hispanic/Latinx	10 (14%)	3 (9%)	4 (10%)
Placement in School			
General Education	31 (42%)	23 (67%)	35 (87%)
Mainstream with Aide	19 (26%)	4 (12%)	0 (0%)
Resource	8 (11%)	4 (12%)	0 (0%)
Special Day	7 (9%)	1 (3%)	0 (0%)
Other	8 (11%)	0 (0%)	2 (5%)
Missing	1 (1%)	2 (6%)	3 (8%)
Time in General Education			
81–100%	48 (65%)	27 (79%)	36 (90%)
61–80%	6 (8%)	3 (9%)	1 (3%)
41–60%	5 (7%)	0 (0%)	1 (3%)
< 40%	7 (9%)	2 (6%)	1 (3%)
None	7 (9%)	2 (6%)	0 (0%)
Missing	1 (1%)	0 (0%)	1 (3%)

**Table 2:** Correlations of SP2 auditory processing and SCAN subtests in all diagnostic groups

SP2 auditory processing	SCAN-3 auditory composite	SCAN-3 filter words	SCAN-3 competing words	SCAN-3 competing sentences	SCAN-3 auditory figure ground

Autism	−.39**	−.22	−.39**	−.42**	−.16
ADHD	−.18	−.03	−.17	.003	−.13
NT	−.04	−.14	.05	.27	−.09

*ADHD* attention deficit/hyperactivity disorder, *NT* neurotypical, *SP2* Sensory Profile, Second Edition

* *p* < .05,

** *p* < .01,

*** *p* < .001

**Table 3:** Multiple regression results for reading variables in the autism group

	Variables	Model 1		Model 2		Model 3		Model 4	
		β	SE	β	SE	β	SE	β	SE

GORT5 reading fluency	WASI-II FIQ	0.53***	0.10	0.40***	0.11	0.36***	0.12	0.37**	0.11
	SCAN-3 Competing Sentences			0.25*	0.11	0.25*	0.12	0.22	0.15
	SP2 Auditory Processing					−0.17	0.12	−0.16	0.12
	SCAN-3 Competing Words							0.06	0.15
	Model Adjusted R^2^	0.30		0.35		0.39		0.38	
	Model F value	27.06		17.09		11.90		8.79	
	Model p-value	< .001		< .001		< .001		< .001	

	Variables	Model 1		Model 2		Model 3			
		β	SE	β	SE	β	SE		

GORT5 reading comprehension	WASI-II FIQ	0.63***	0.09	0.52***	0.11	0.51***	0.12		
	SCAN-3 Competing Sentences			0.15	0.11	0.11	0.12		
	SP2 Auditory Processing					−0.17	0.12		
	Model Adjusted R2	0.40		0.40		0.40			
	Model F Value	45.33		20.72		12.53			
	Model p-value	< .001		< .001		< .001			

WASI-II = Wechsler Abbreviated Scale of Intelligence, Second Edition, FIQ = Full-scale IQ, GORT-5 = Gray Oral Reading Test, Fifth Edition. Regression analyses were based on the full sample of autistic individuals (N = 74) which included 22 children with a co-occurring diagnosis of ADHD

* *p* < .05,

** *p* < .01,

*** *p* < .001

**Table 4:** Multiple regression results for the mathematics variables in the autism group

	Variables	Model 1		Model 2		Model 3		Model 4	
		β	SE	β	SE	β	SE	β	SE

WIAT-III problem solving	FIQ	0.75***	0.09	0.69***	0.09	0.72***	0.08	0.72***	0.10
	SCAN-3 Filtering Words			0.25**	0.09	0.28**	0.09	0.28*	0.11
	SCAN-3 Competing Sentences					−0.07	0.10	−0.07	0.10
	SCAN-3 Auditory Figure Ground							0.002	0.11
	Model Adjusted R^2^	0.54		0.59		0.60		0.59	
	Model F Value	22.43		46.04		30.44		22.43	
	Model p-value	< .001		< .001		< .001		< .001	

	Variables	Model 1		Model 2					
		β	SE	β	SE				

WIAT-III numerical operations	FIQ	0.58***	0.10	0.56***	0.13				
	SCAN-3 Competing Sentences			0.04	0.12				
	Model Adjusted R^2^	0.33		0.31					
	Model F Value	33.86		14.57					
	Model p-value	< .001		< .001					

WASI-II = Wechsler Abbreviated Scale of Intelligence, Second Edition, FIQ = Full-scale IQ, WIAT-III = Wechsler Individual Achievement Test, Third Edition, SP2 = Sensory Profile, Second Edition. Regression analyses were based on the full sample of autistic individuals (N = 74) which included 22 children with a co-occurring diagnosis of ADHD

* *p* < .05,

** *p* < .01,

*** *p* < .001

**Table 5:** Multiple regression results for the reading variables in the ADHD group

	Variables	Model 1				Model 2	
		β		SE		β	SE

GORT5 reading fluency	FIQ	0.39***		0.17		0.18	0.16
	SCAN-3 Competing Sentences					0.49**	0.16
	Model Adjusted R^2^	0.13				0.32	
	Model F value	5.28				7.86	
	Model p-value	.03				0.002	

	Variables	Model 1		Model 2		Model 3	
		β	SE	β	SE	β	SE

GORT5 reading comprehension	FIQ	0.67***	0.14	0.46***	0.12	0.45**	0.12
	SCAN-3 Competing Sentences			0.49***	0.12	0.48***	0.13
	SCAN-3 Competing Words					0.04	0.14
	Model Adjusted R2	0.45		0.66		0.65	
	Model F Value	25.00		29.07		18.75	
	Model p-value	< .001		< .001		< .001	

WASI-II = Wechsler Abbreviated Scale of Intelligence, Second Edition, FIQ = Full-scale IQ, GORT-5 = Gray Oral Reading Test, Fifth Edition. Regression analyses included the 34 non-autistic children with ADHD

* *p* < .05,

** *p* < .01,

*** *p* < .001

**Table 6:** Multiple regression results for mathematics variables in the ADHD group

	Variables	Model 1		Model 2		Model 3		Model 4	
		β	SE	β	SE	β	SE	β	SE

WIAT-III problem solving	FIQ	0.77***	0.11	0.72***	0.13	0.69***	0.13	0.64***	0.13
	SCAN-3 Competing Words			0.13	0.13	0.09	0.14	0.11	0.13
	SCAN-3 Competing Sentences					0.10	0.13	0.06	0.13
	SCAN-3 Auditory Figure Ground							0.24	0.11
	Model Adjusted R^2^	0.62		0.62		0.61		0.66	
	Model F Value	47.97		24.40		16.21		15.03	
	Model p-value	< .001		< .001		< .001		< .001	

	Variables	Model 1		Model 2					
		β	SE	β	SE				

WIAT-III numerical operations	FIQ	0.69***	0.13	0.52***	0.11				
	SCAN-3 Competing Sentences			0.41**	0.12				
	Model Adjusted R^2^	0.48		0.61					
	Model F Value	27.66		24.02					
	Model p-value	< .001		< .001					

WASI-II = Wechsler Abbreviated Scale of Intelligence, Second Edition, FIQ = Full-scale IQ, WIAT-III = Wechsler Individual Achievement Test, Third Edition, SP2 = Sensory Profile, Second Edition. Regression analyses included the 34 non-autistic children with ADHD

* *p* < .05,

** *p* < .01,

*** *p* < .001

## References

[R1] Alcantara JI, Weisblatt EJL, Moore BCJ, & Bolton PF (2004). Speech-in-noise perception in high-functioning individuals with autism or Asperger’s syndrome. Journal of Child Psychology and Psychiatry, 45(6), 1107–1114. 10.1111/j.1469-7610.2004.t01-1-00303.x 15257667

[R2] American Psychiatric Association. (2013). Diagnostic and statistical manual of mental disorders (5th ed.). American Psychiatric Association. 10.1176/appi.books.9780890425596

[R3] Armstrong-Gallegos S, & Nicolson RI (2024). Sensory processing abnormalities in school-age children with neurodevelopmental disorders are associated with the range of learning difficulties. International Journal of Disability, Development and Education. 10.1080/1034912X.2024.2370801

[R4] Ashburner J, Ziviani J, & Rodger S (2008). Sensory processing and classroom emotional, behavioral, and educational outcomes in children with autism spectrum disorder. American Journal of Occupational Therapy, 62(5), 564–573. 10.5014/ajot.62.5.564 18826017

[R5] Baranek GT, David FJ, Poe MD, Stone WL, & Watson LR (2006). Sensory experiences questionnaire: Discriminating sensory features in young children with autism, developmental delays, and typical development. Journal of Child Psychology and Psychiatry, 47(6), 591–601. 10.1111/j.1469-7610.2005.01546.x 16712636

[R6] Baranek GT, Watson LR, Boyd BA, Poe MD, David FJ, & McGuire L (2013). Hyporesponsiveness to social and nonsocial sensory stimuli in children with autism, children with developmental delays, and typically developing children. Development and Psychopathology, 25(2), 307–320. 10.1017/S0954579412001071 23627946 PMC3641693

[R7] Berument SK, Rutter M, Lord C, Pickles A, & Bailey A (1999). Autism screening questionnaire: Diagnostic validity. British Journal of Psychiatry, 175(5), 444–451. 10.1192/bjp.175.5.444 10789276

[R8] Bijlenga D, Tjon-Ka-Jie JYM, Schuijers F, & Kooij JJS (2017). Atypical sensory profiles as core features of adult ADHD, irrespective of autistic symptoms. European Psychiatry, 43, 51–57. 10.1016/j.eurpsy.2017.02.481 28371743

[R9] Bullen JC, Swain Lerro L, Zajic M, McIntyre N, & Mundy P (2020). A developmental study of mathematics in children with autism spectrum disorder, symptoms of attention deficit hyperactivity disorder, or typical development. Journal of Autism and Developmental Disorders, 50(12), 4463–4476. 10.1007/s10803-020-04500-9 32306219

[R10] Butera C, Ring P, Sideris J, Jayashankar A, Kilroy E, Harrison L, Cermak S, & Aziz-Zadeh L (2020). Impact of sensory processing on school performance outcomes in high functioning individuals with autism spectrum disorder. Mind, Brain, and Education, 14(3), 243–254. 10.1111/mbe.12242 PMC8341443 34367324

[R11] Cacace AT, & McFarland DJ (2006). Delineating auditory processing disorder (APD) and attention deficit hyperactivity disorder (ADHD): A conceptual, theoretical, and practical framework. In Parthasarathy TK (Ed.), An introduction to auditory processing disorders in children (1st ed., pp. 39–61). Psychology Press.

[R12] Chang YS, Owen JP, Desai SS, Hill SS, Arnett AB, Harris J, & Mukherjee P (2014). Autism and sensory processing disorders: Shared white matter disruption in sensory pathways but divergent connectivity in social-emotional pathways. PLoS ONE, 9(7), 1–17. 10.1371/journal.pone.0103038 PMC4116166 25075609

[R13] Chermak GD, Hall JW, & Musiek FE (1999). Differential diagnosis and management of central auditory processing disorder and attention deficit hyperactivity disorder. Journal of the American Academy of Audiology, 10(6), 289–303. 10385872

[R14] Cheung PPP, & Siu AMH (2009). A comparison of patterns of sensory processing in children with and without developmental disabilities. Research in Developmental Disabilities, 30(6), 1468–1480. 10.1016/j.ridd.2009.07.009 19665348

[R15] Classi P, Milton D, Ward S, Sarsour K, & Johnston J (2012). Social and emotional difficulties in children with ADHD and the impact on school attendance and healthcare utilization. Child and Adolescent Psychiatry and Mental Health, 6(33), 1–8. 10.1186/1753-2000-6-33 23035861 PMC3489829

[R16] Conners KC (2008). Conners 3rd edition. Multi-Health Systems.

[R17] Constantino JN, Davis SA, Todd RD, Schindler MK, Gross MM, Brophy SL, Metzger LM, Shoushtari CS, Splinter R, & Reich W (2003). Validation of a brief quantitative measure of autistic traits: Comparison of the social responsiveness scale with the autism diagnostic interview-revised. Journal of Autism and Developmental Disorders, 33(4), 427–433. 10.1023/A:1025014929212 12959421

[R18] Constantino JN, Przybeck T, Friesen D, & Todd RD (2000). Reciprocal social behavior in children with and without pervasive developmental disorders. Journal of Developmental and Behavioral PediatriCs, 21(1), 2–11. 10706343 10.1097/00004703-200002000-00002

[R19] Craddock E (2024). Raising the voices of AuDHD women and girls: Exploring the co-occurring conditions of autism and ADHD. Disability & Society, 39(8), 2161–2165. 10.1080/09687599.2023.2299342

[R20] Davis AS, Pass LA, Finch WH, Dean RS, & Woodcock RW (2009). The canonical relationship between sensory-motor functioning and cognitive processing in children with attention-deficit/hyperactivity disorder. Archives of Clinical Neuropsychology, 24(3), 273–286. 10.1093/arclin/acp032 19574293

[R21] Dawes P, & Bishop D (2009). Auditory processing disorder in relation to developmental disorders of language, communication and attention: A review and critique. International Journal of Language & Communication Disorders, 44(4), 440–465. 10.1080/13682820902929073 19925352

[R22] De la Marche W, Steyaert J, & Noens I (2012). Atypical sensory processing in adolescents with an autism spectrum disorder and their non-affected siblings. Research in Autism Spectrum Disorders, 6(2), 639–645. 10.1016/j.rasd.2011.09.014

[R23] Dellapiazza F, Michelon C, Oreve M-J, Robel L, Schoenberger M, Chatel C, Vesperini S, Maffre T, Schmidt R, Blanc N, Vernhet C, Picot M-C, & Baghdadli A (2020). The impact of atypical sensory processing on adaptive functioning and maladaptive behaviors in autism spectrum disorder during childhood: Results from the ELENA cohort. Journal of Autism and Developmental Disorders, 50(6), 2142–2152. 10.1007/s10803-019-03970-w 30868365

[R24] Dellapiazza F, Vernhet C, Blanc N, Miot S, Schmidt R, & Baghdadli A (2018). Links between sensory processing, adaptive behaviours, and attention in children with autism spectrum disorder: A systematic review. Psychiatry Research, 270, 78–88. 10.1016/j.psychres.2018.09.023 30245380

[R25] Demopoulos C, & Lewine JD (2016). Audiometric profiles in autism spectrum disorders: Does subclinical hearing loss impact communication? Autism Research, 9(1), 107–120. 10.1002/aur.1495 25962745 PMC4641833

[R26] DePape AMR, Hall GB, Tillmann B, & Trainor LJ (2012). Auditory processing in high-functioning adolescents with autism spectrum disorder. PLoS ONE. 10.1371/journal.pone.0044084 PMC3440400 22984462

[R27] Dunn W (1997). The impact of sensory processing abilities on the daily lives of young children and their families: A conceptual model. Infants & Young Children, 9(4), 23–35. 10.1097/00001163-199704000-00005

[R28] Dunn W (1999). The sensory profile. The Psychological Corporation.

[R29] Dunn W (2014). Sensory profile-2. Pearson Publishing.

[R30] Dunn W, & Bennett D (2002). Patterns of sensory processing in children with attention deficit hyperactivity disorder. OTJR, Occupational Therapy Journal of Research, 22(1), 4–15. 10.1177/153944920202200102

[R31] Ehlers S, Gillberg C, & Wing L (1999). A screening question-naire for Asperger syndrome and other high-functioning autism spectrum disorders in school age children. Journal of Autism and Developmental Disorders, 29(2), 129–141. 10.1023/A:1023040610384 10382133

[R32] Fernández-Andrés MI, Pastor-Cerezuela G, Sanz-Cervera P, & Tárraga-Mínguez R (2015). A comparative study of sensory processing in children with and without autism spectrum disorder in the home and classroom environments. Research in Developmental Disabilities, 38, 202–212. 10.1016/j.ridd.2014.12.034 25575284

[R33] Font-Alaminos M, Cornella M, Costa-Faidella J, Hervás A, Leung S, Rueda I, & Escera C (2020). Increased subcortical neural responses to repeating auditory stimulation in children with autism spectrum disorder. Biological Psychology, 149, 1–8. 10.1016/j.biopsycho.2019.107807 31693923

[R34] Ghanizadeh A (2011). Sensory processing problems in children with ADHD, a systematic review. Psychiatry Investigation, 8(2), 89. 10.4306/pi.2011.8.2.89 21852983 PMC3149116

[R35] Groen WB, van Orsouw L, ter Huurne N, Swinkels S, van der Gaag R-J, Buitelaar JK, & Zwiers MP (2009). Intact spectral but abnormal temporal processing of auditory stimuli in autism. Journal of Autism and Developmental Disorders, 39(5), 742–750. 10.1007/s10803-008-0682-3 19148738

[R36] Hanley M, Khairat M, Taylor K, Wilson R, Cole-Fletcher R, & Riby DM (2017). Classroom displays—Attraction or distraction? Evidence of impact on attention and learning from children with and without autism. Developmental Psychology, 53(7), 1265–1275. 10.1037/DEV0000271 28471220

[R37] Hochhauser M, & Engel-Yeger B (2010). Sensory processing abilities and their relation to participation in leisure activities among children with high-functioning autism spectrum disorder (HFASD). Research in Autism Spectrum Disorders, 4(4), 746–754. 10.1016/j.rasd.2010.01.015

[R38] Howe FE, & Stagg SD (2016). How sensory experiences affect adolescents with an autistic spectrum condition within the classroom. Journal of Autism and Developmental Disorders, 46, 1656–1668. 10.1007/s10803-015-2693-1 26791372 PMC4826419

[R39] Hulslander J, Talcott J, Witton C, DeFries J, Pennington B, Wadsworth S, Willcutt E, & Olson R (2004). Sensory processing, reading, IQ, and attention. Journal of Experimental Child Psychology, 88(3), 274–295. 10.1016/j.jecp.2004.03.006 15203301

[R40] Irwin V, De La Rosa J, Wang K, Hein S, Zhang J, Burr R, Roberts A, Barmer A, Bullock Mann F, Dilig R, & Parker S (2022). Report on the condition of education 2022 (NCES 2022144). U.S. Department of Education. Washington, DC: National Center for Education Statistics. Retrieved from https://nces.ed.gov/pubsearch/pubsinfo.asp?pubid=2022144

[R41] Jasmin E, Couture M, McKinley P, Reid G, Fombonne E, & Gisel E (2009). Sensori-motor and daily living skills of preschool children with autism spectrum disorders. Journal of Autism and Developmental Disorders, 39(2), 231–241. 10.1007/s10803-008-0617-z 18629623

[R42] Jones E, Hanley M, Hirst J, Mc Dougal E, & Riby DM (2024). The effect of the classroom sensory environment on engagement for autistic pupils: Classroom noise, classroom displays, and teacher display practices. OSF. 10.31219/osf.io/2ktgf

[R43] Jones EK, Hanley M, & Riby DM (2020). Distraction, distress and diversity: Exploring the impact of sensory processing differences on learning and school life for pupils with autism spectrum disorders. Research in Autism Spectrum Disorders, 72, Article 101515. 10.1016/j.rasd.2020.101515

[R44] Jones RSP, Quigney C, & Huws JC (2003). First-hand accounts of sensory-perceptual experiences in autism: A qualitative analysis. Journal of Intellectual and Developmental Disability, 28(2), 112–121. 10.1080/1366825031000147058

[R45] Keen D, Webster A, & Ridley G (2016). How well are children with autism spectrum disorder doing academically at school? An overview of the literature. Autism, 20(3), 276–294. 10.1177/1362361315580962 25948598

[R46] Keith JM, Jamieson JP, & Bennetto L (2019). The importance of adolescent self-report in autism spectrum disorder: Integration of questionnaire and autonomic measures. Journal of Abnormal Child Psychology, 47, 741–754. 10.1007/s10802-018-0455-1 30073571 PMC6359986

[R47] Keith RW (2000). Development and standardization of SCAN-C test for auditory processing disorders in children. Journal of the American Academy of Audiology. 10.1055/s-0042-1748131 11012239

[R48] Keith RW (2009). SCAN-3 for adolescents and adults: Tests for auditory processing disorders. Pearson.

[R49] Khalfa S, Bruneau N, Rogé B, Georgieff N, Veuillet E, Adrien J-L, Barthélémy C, & Collet L (2004). Increased perception of loudness in autism. Hearing Research, 198(1–2), 87–92. 10.1016/j.heares.2004.07.006 15617227

[R50] Kim SH, Bal VH, & Lord C (2018). Longitudinal follow-up of academic achievement in children with autism from age 2 to 18. Journal of Child Psychology and Psychiatry, 59(3), 258–267. 10.1111/jcpp.12808 28949003 PMC5819744

[R51] Konold TR, & Canivez GL (2010). Differential relationships between WISC-IV and WIAT-II scales: An evaluation of potentially moderating child demographics. Educational and Psychological Measurement, 70(4), 613–627.

[R52] Leekam SR, Nieto C, Libby SJ, Wing L, & Gould J (2007). Describing the sensory abnormalities of children and adults with autism. Journal of Autism and Developmental Disorders, 37(5), 894–910. 10.1007/s10803-006-0218-7 17016677

[R53] Leitner Y (2014). The co-occurrence of autism and attention deficit hyperactivity disorder in children–what do we know? Frontiers in Human Neuroscience, 8, 268. 10.3389/fnhum.2014.00268 24808851 PMC4010758

[R54] Liss M, Saulnier C, Fein D, & Kinsbourne M (2006). Sensory and attention abnormalities in autistic spectrum disorders. Autism, 10(2), 155–172. 10.1177/1362361306062021 16613865

[R55] Little LM, Dean E, Tomchek S, & Dunn W (2018). Sensory processing patterns in autism, attention deficit hyperactivity disorder, and typical development. Physical & Occupational Therapy in Pediatrics, 38(3), 243–254. 10.1080/01942638.2017.1390809 29240517

[R56] Lord C, Rutter M, DiLavore P, Risi S, Gotham K, & Bishop S (2012). Autism diagnostic observation schedule (2nd ed.). Western Psychological Services.

[R57] Mallory C, & Keehn B (2021). Implications of sensory processing and attentional differences associated with autism in academic settings: An integrative review. Frontiers in Psychiatry, 12, Article 695825. 10.3389/fpsyt.2021.695825 34512416 PMC8430329

[R58] McIntyre NS, Solari EJ, Grimm RP, Lerro E, L., E. Gonzales, J., & Mundy, P. C. (2017). A comprehensive examination of reading heterogeneity in students with high functioning autism: Distinct reading profiles and their relation to autism symptom severity. Journal of Autism and Developmental Disorders, 47(4), 1086–1101. 10.1007/s10803-017-3029-0 28160222

[R59] McLaughlin CS, Grosman HE, Guillory SB, Isenstein EL, Wilkinson E, Trelles MdelP, Halpern DB, Siper PM, Kolevzon A, Buxbaum JD, Wang AT, & Foss-Feig JH (2021). Reduced engagement of visual attention in children with autism spectrum disorder. Autism, 25(7), 2064–2073. 10.1177/13623613211010072 33966481 PMC8547710

[R60] Miller LJ, Anzalone ME, Lane SJ, Cermak SA, & Osten ET (2007). Conceptual evolution in sensory integration: A proposed nosology for diagnosis. American Journal of Occupational Therapy, 61(2), 135–140. https://sensoryhealth.org/sites/default/files/publications/conceptevolutioninsensoryintegration.pdf 10.5014/ajot.61.2.135 17436834

[R61] Moore DR, Ferguson MA, Edmondson-Jones AM, Ratib S, & Riley A (2010). Nature of auditory processing disorder in children. Pediatrics, 126(2), e382–e390. 10.1542/peds.2009-2826 20660546

[R62] Narzisi A, Fabbri-Destro M, Crifaci G, Scatigna S, Maugeri F, Berloffa S, Fantozzi P, Prato A, Muccio R, Valente E, Viglione V, Pecchini E, Pelagatti S, Rizzo R, Milone A, Barone R, & Masi G (2022). Sensory profiles in school-aged children with autism spectrum disorder: A descriptive study using the Sensory Processing Measure-2 (SPM-2). Journal of Clinical Medicine, 11(6), 1668. 10.3390/jcm11061668 35329994 PMC8955781

[R63] O’Connor K (2012). Auditory processing in autism spectrum disorder: A review. Neuroscience & Biobehavioral Reviews, 36(2), 836–854. 22155284 10.1016/j.neubiorev.2011.11.008

[R64] Puts NAJ, Wodka EL, Harris AD, Crocetti D, Tommerdahl M, Mostofsky SH, & Edden RAE (2017). Reduced GABA and altered somatosensory function in children with autism spectrum disorder: Abnormal GABA and touch in ASD. Autism Research, 10(4), 608–619. 10.1002/aur.1691 27611990 PMC5344784

[R65] Puts NAJ, Wodka EL, Tommerdahl M, Mostofsky SH, & Edden RAE (2014). Impaired tactile processing in children with autism spectrum disorder. Journal of Neurophysiology, 111(9), 1803–1811. 10.1152/jn.00890.2013 24523518 PMC4044368

[R66] Rutter M, Bailey A, & Lord C (2003). Manual for the social communication questionnaire. Western Psychological Services.

[R67] Sanz-Cervera P, Pastor-Cerezuela G, Fernández-Andrés M-I, & Tárraga-Mínguez R (2015). Sensory processing in children with autism spectrum disorder: Relationship with non-verbal IQ, autism severity and attention deficit/hyperactivity disorder symptomatology. Research in Developmental Disabilities, 45–46, 188–201. 10.1016/j.ridd.2015.07.031 26263405

[R68] Sasson NJ (2006). The development of face processing in autism. Journal of Autism and Developmental Disorders, 36(3), 381–394. 10.1007/s10803-006-0076-3 16572261

[R69] St. John T, Estes A, Begay KK, Munson J, Reiter MA, Dager SR, & Kleinhans N (2022). Characterizing social functioning in school-age children with sensory processing abnormalities. Journal of Autism and Developmental Disorders, 52(3), 1361–1373. 10.1007/s10803-021-05050-4 33956254 PMC8854314

[R70] Stevens T, Peng L, & Barnard-Brak L (2016). The comorbidity of ADHD in children diagnosed with autism spectrum disorder. Research in Autism Spectrum Disorders, 31, 11–18. 10.1016/j.rasd.2016.07.003

[R71] Tomchek SD, & Dunn W (2007). Sensory processing in children with and without autism: A comparative study using the short sensory profile. American Journal of Occupational Therapy, 61(2), 190–200. 10.5014/ajot.61.2.190 17436841

[R72] Vlaskamp C, Oranje B, Madsen GF, Møllegaard Jepsen JR, Durston S, Cantio C, & Bilenberg N (2017). Auditory processing in autism spectrum disorder: Mismatch negativity deficits. Autism Research, 10(11), 1857–1865. 10.1002/aur.1821 28639417

[R73] Wechsler D (2009). Wechsler individual achievement test, (WIAT-III) (3rd ed.). The Psychological Corporation.

[R74] Wechsler D (2011). Wechsler abbreviated scale of intelligence (2nd ed.). NCS Pearson.

[R75] Werkman MF, Landsman JA, Fokkens AS, Dijkxhoorn YM, van Berckelaer-Onnes IA, Begeer S, & Reijneveld SA (2022). The impact of the presence of intellectual disabilities on sensory processing and behavioral outcomes among individuals with autism spectrum disorders: A systematic review. Review Journal of Autism and Developmental Disorders. 10.1007/s40489-022-00301-1

[R76] Wiederholt JL, & Bryant BR (2012). Gray oral reading tests—Fifth edition (GORT-5). Pro-Ed.

[R77] Yochman A, Parush S, & Ornoy A (2004). Responses of preschool children with and without adhd to sensory events in daily life. American Journal of Occupational Therapy, 58(3), 294–302. 10.5014/ajot.58.3.294 15202627

[R78] Zajic MC, Solari EJ, McIntyre NS, Lerro L, & Mundy PC (2020). Task engagement during narrative writing in school-age children with autism spectrum disorder compared to peers with and without attentional difficulties. Research in Autism Spectrum Disorders, 76, Article 101590. 10.1016/j.rasd.2020.101590

