# Supplementary material for: Sensory Processing Modalities and Their Associations With Academic Achievement in Autism and Attention Deficit/Hyperactivity Disorder
Source: J Autism Dev Disord. Author manuscript; Available in PMC 2026 Jun 5. (PMC13241175; doi:10.1007/s10803-025-07185-0)
Supplement: Supplementary Material [file NIHMS2169297-supplement-Supplementary_Material.docx]

**Supplementary Table 1.**

*Correlations of Sensory Processing, FIQ, and Academic Achievement Variables in the Autism Group*

|  | **WASI-II FIQ** | **GORT-5 Fluency** | **GORT-5 Comprehension** | **WIAT-III Numerical Operations** | **WIAT-III Problem Solving** |
| --- | --- | --- | --- | --- | --- |
| **SP2 Auditory** | -.30* | -.39* | -.32* | -.16 | -.25 |
| **SP2 Visual** | .05 | .06 | -.08 | -.10 | -.02 |
| **SP2 Touch** | .06 | -.11 | -.05 | -.04 | .02 |
| **SCAN-3 Filter Words** | .29** | .13 | .13 | .18 | .42*** |
| **SCAN-3 Competing Words** | .25* | .36** | .24 | .08 | .23 |
| **SCAN-3 Competing Sentences** | .47*** | .48*** | .43*** | .32*** | .40** |
| **SCAN-3 Auditory Figure Ground** | .33** | .19 | .16 | .10 | .37** |
| **SCAN-3 Auditory Composite** | .48*** | .40** | .35*** | .29* | .48*** |

*Note.* * *p* < .05, ** *p* < .01, *** *p* < .001. WASI-II = Wechsler Abbreviated Scale of Intelligence, Second Edition, FIQ = Full-scale IQ, GORT-5 = Gray Oral Reading Test, Fifth Edition, WIAT-III = Wechsler Individual Achievement Test, Third Edition, SP2 = Sensory Profile, Second Edition. Correlation analyses were conducted on the full autism sample (N = 74) which included 22 individuals with a co-occurring diagnosis of ADHD.

**Supplementary Table 2.**

*Correlations of Sensory Processing, FIQ, and Academic Achievement Variables in the ADHD Group*

|  | **WASI-II FIQ** | **GORT-5 Fluency** | **GORT-5 Comprehension** | **WIAT-III Numerical Operations** | **WIAT-III Problem Solving** |
| --- | --- | --- | --- | --- | --- |
| **SP2 Auditory** | .07 | .27 | .05 | .04 | -.07 |
| **SP2 Visual** | .30 | .11 | .13 | .12 | .18 |
| **SP2 Touch** | .48* | .13 | .09 | .09 | .19 |
| **SCAN-3 Filter Words** | .09 | .32 | .37* | .11 | .05 |
| **SCAN-3 Competing Words** | .42* | .14 | .48** | .31 | .46* |
| **SCAN-3 Competing Sentences** | .32 | .58*** | .71*** | .64*** | .44* |
| **SCAN-3 Auditory Figure Ground** | .22 | .14 | .20 | .43* | .45* |
| **SCAN-3 Auditory Composite** | .39* | .49** | .66*** | .57** | .45* |

*Note.* * *p* < .05, ** *p* < .01, *** *p* < .001. ADHD = attention deficit/hyperactivity disorder, WASI-II = Wechsler Abbreviated Scale of Intelligence, Second Edition, FIQ = Full-scale IQ, GORT-5 = Gray Oral Reading Test, Fifth Edition, WIAT-III = Wechsler Individual Achievement Test, Third Edition, SP2 = Sensory Profile, Second Edition. Correlation analyses were conducted on the sample of 34 non-autistic children with ADHD.

**Supplementary Table 3.**

*Correlations of sensory processing, FIQ, academic achievement variables in the NT Group.*

|  | **WASI-II FIQ** | **GORT-5 Fluency** | **GORT-5 Comprehension** | **WIAT-III Numerical Operations** | **WIAT-III Problem Solving** |
| --- | --- | --- | --- | --- | --- |
| **SP2 Auditory** | -.35 | -.35 | -.25 | -.06 | -.01 |
| **SP2 Visual** | -.12 | -.25 | -.07 | -.02 | .06 |
| **SP2 Touch** | -.12 | -.05 | .15 | -.05 | .03 |
| **SCAN-3 Filter Words** | -.22 | -.05 | -.14 | -.23 | -.19 |
| **SCAN-3 Competing Words** | .08 | -.19 | -.02 | -.02 | -.21 |
| **SCAN-3 Competing Sentences** | -.01 | .18 | .24 | .03 | .03 |
| **SCAN-3 Auditory Figure Ground** | .15 | .04 | .03 | .25 | .21 |
| **SCAN-3 Auditory Composite** | .04 | .20 | .15 | .07 | .07 |

*Note.* * *p* < .05, ** *p* < .01, *** *p* < .001. NT = neurotypical, WASI-II = Wechsler Abbreviated Scale of Intelligence, Second Edition, FIQ = Full-scale IQ, GORT-5 = Gray Oral Reading Test, Fifth Edition, WIAT-III = Wechsler Individual Achievement Test, Third Edition, SP2 = Sensory Profile, Second Edition.

|  |  | Model 1 | | Model 2 | | Model 3 | | Model 4 | |
| --- | --- | --- | --- | --- | --- | --- | --- | --- | --- |
|  | Variables | β | SE | β | SE | β | SE | β | SE |
| *GORT5 Reading Fluency* | WASI-II FIQ | 0.53*** | 0.10 | 0.43*** | 0.12 | 0.43** | 0.12 | 0.42** | 0.13 |
|  | SCAN-3 Competing Sentences |  |  | 0.24* | 0.12 | 0.26* | 0.13 | 0.33 | 0.16 |
|  | SP2 Auditory Processing |  |  |  |  | -0.15 | 0.12 | -0.17 | 0.13 |
|  | SCAN-3 Competing Words |  |  |  |  |  |  | -0.11 | 0.16 |
|  | Model Adjusted R^2^ | 0.35 | | 0.39 | | 0.44 | | 0.44 | |
|  | Model F value | 23.85 | | 14.70 | | 10.59 | | 7.94 | |
|  | Model p-value | < .001 | | < .001 | | < .001 | | < .001 | |
|  |  | Model 1 | | Model 2 | | Model 3 | |  | |
|  | Variables | β | SE | β | SE | β | SE |  |  |
| *GORT5 Reading Comprehension* | WASI-II FIQ | 0.63*** | 0.11 | 0.50*** | 0.11 | 0.52*** | 0.13 |  |  |
|  | SCAN-3 Competing Sentences |  |  | 0.20 | 0.12 | 0.21 | 0.13 |  |  |
|  | SP2 Auditory Processing |  |  |  |  | -0.04 | 0.13 |  |  |
|  | Model Adjusted R2 | 0.43 | | 0.43 | | 0.42 | |  |  |
|  | Model F Value | 35.78 | | 17.41 | | 9.54 | |  |  |
|  | Model p-value | < .001 | | < .001 | | < .001 | |  |  |

**Supplementary Table 4.**

*Multiple Regression Results for Reading Variables in the Autism Group excluding individuals with a diagnosis of ADHD.*

*Note.* * *p* < .05, ** *p* < .01, *** *p* < .001. WASI-II = Wechsler Abbreviated Scale of Intelligence, Second Edition, FIQ = Full-scale IQ, GORT-5 = Gray Oral Reading Test, Fifth Edition. Regression analyses were conducted on the 52 autistic children who did not have a co-occurring diagnosis of ADHD (N = 22).

**Supplementary Table 5.**

*Multiple Regression Results for the Mathematics Variables in the Autism Group excluding individuals with a diagnosis of ADHD.*

|  |  | Model 1 | | | Model 2 | | Model 3 | | | Model 4 | |
| --- | --- | --- | --- | --- | --- | --- | --- | --- | --- | --- | --- |
|  | Variables | β | SE | | β | SE | | β | SE | β | SE |
| *WIAT-III Problem Solving* | FIQ | 0.72*** | 0.10 | | 0.69*** | 0.10 | | 0.72*** | 0.11 | 0.72*** | 0.11 |
|  | SCAN-3 Filtering Words |  |  | | 0.22* | 0.11 | | 0.25* | 0.11 | 0.24 | 0.13 |
|  | SCAN-3 Competing Sentences |  |  | |  |  | | -0.09 | 0.12 | -0.10 | 0.12 |
|  | SCAN-3 Auditory Figure Ground |  |  | |  |  | |  |  | 0.01 | 0.13 |
|  | Model Adjusted R^2^ | 0.53 | | | 0.56 | | 0.57 | | | 0.56 | |
|  | Model F Value | 51.21 | | | 29.52 | | 19.81 | | | 14.49 | |
|  | Model p-value | < .001 | | | < .001 | | < .001 | | | < .001 | |
|  |  | Model 1 | | | Model 2 | |  | |  |  |  |
|  | Variables | β | | SE | β | SE | |  |  |  |  |
| *WIAT-III Numerical Operations* | FIQ | 0.59*** | | 0.12 | 0.56*** | 0.13 | |  |  |  |  |
|  | SCAN-3 Competing Sentences |  | |  | 0.05 | 0.14 | |  |  |  |  |
|  | Model Adjusted R^2^ | 0.36 | | | 0.33 | |  | |  |  |  |
|  | Model F Value | 27.25 | | | 11.77 | |  | |  |  |  |
|  | Model p-value | < .001 | | | < .001 | |  | |  |  |  |

*Note.* * *p* < .05, ** *p* < .01, *** *p* < .001. WASI-II = Wechsler Abbreviated Scale of Intelligence, Second Edition, FIQ = Full-scale IQ, WIAT-III = Wechsler Individual Achievement Test, Third Edition, SP2 = Sensory Profile, Second Edition. Regression analyses were conducted on the 52 autistic children who did not have a co-occurring diagnosis of ADHD (N = 22).
